# Prescription Department

**Published:** 1892-10

**Authors:** 


Rheumatic Bronchitis.—By *Dr. N. S. Davis.*

℞. Sodii salicylatis, dr. vi.
Glycerinæ, dr. iv.
Vini colch. rad., dr. vi.
Syr. scillæ comp., dr. iss.
Tr. opii camph., oz. ij.

M. S. A teaspoonful every three or four hours, in a little water.

Pruritus Ani and Vulvæ.—

The following formula will afford relief from the itching and irritation—to be applied locally:

℞. Sodii hyposulphis, dr. i.

Acid carbol., dr. ss.

Glycerinæ, oz. i.

Listerine, oz. iii. M.

Hepatic Eruptions or "Cold Sores" on Lips.—By *Dr. R. L. Patterson*, Bridgeville, Pa.

℞. Spir. camphor, dr. vj.
Liniment camphor, U. S.P., oz. j. (Camphorated oil).
Declat's glyco. phenique, dr. ij.

M. S. Apply frequently.

Typhoid Fever.—By *Emil Bories*, Dayton, Wash.

℞. Eucalyptol, (Sander's), dr. iij.
Muc. acaciæ, oz. ij.
Saccharine, gr. iij.
Sodæ bicarbonas, gr. vj.
Aquæ. distil. q. s. ad., oz. iv.

M. S. Teaspoonful every three hours.

A Salve for Hemorrhoids.—*Dr. Rosobudsky* (*Munch. med. Woch.*, No. 35, 1892) praises the following:

℞. Chrysarobin, gms. 0.8 (grs. xij).

Iodoform, gms. 0.3 (grs. v).

Extract of belladonna, gms. 0.6 (grs. ix).

Vaseline, gms. 15 (dr. iv).

—*Ex.*

Acute Gout.—By *Dr. A. L. Loomis.*

℞. Pulv. ipecac, gr. i.
Ext. colchi acet, gr. i.
Calomel, gr. i.
Ext. aloes fl., gr i.
Ext. nux. vom., gr. ¼.

Ft. pil. S. One every three hours until the specific purgative action of colchicum is obtained.

Acute Gastritis.—By *Prof. Pepper*, Philadelphia, Pa.

1. Absolute rest for stomach.
2. Nutritive enemata.
3. To quiet stomach.

R. Hydrarg. chlor. mitis., gr. ij.
Bismuthi subnit. dr. j.

M. Ft. chart. No. xx. S. One powder every four hours.

Or:

R. Acid. carbolic, gtt. iv.
Sodii bicarb., dr. jss.
Elixir, f oz. ss.
Aquæ, q. s. ad. f. oz. iv.

M. S. Dr. j every three hours.

4. Opium, hyoscyamus, asafœtida by suppository for nervous symptoms.
5. Counter irritation over epigastrium.

Give nothing by mouth except for its local action on the stomach.

Especially avoid all purges. If one is necessary use calomel.

Antonic Dyspepsia.—

R. Zinci valerianatis, dr. ss.
Ext. belladonnæ, gr. iij.
Ext. nucis vomicæ, gr. v.

M. Ft. pil. No. xxx. S. One pill after each meal.

Vomiting of Pregnancy.—*Dr. Frank R. Fry* (*Times and Register*) recommends the following:

R. Cocain. muriat. grs. vj.
Cerii oxalat, grs. xxiv.
Glycerine,
Aqua laurocer, aa dr. iij.
Aqua destil., q. s. oz. iij.

M. ft. mist. S. One tablespoonful (taken cold) every two hours once or twice a day, pro re nata.

Tonic after a Debauch.—

*Dr. Whitla* says that the following is of service in cases in which insomnia, anorexia, loss of will power, and mild hallucinations, following a long debauch, point to threatening delirium tremens:

R. Quinæ sulphatis, grs. xxv.
Acidi nitrohydrochlor. dil., dr. vi.
Ext. cinchonæ, dr. iij.
Aquæ destillatæ, q. s., ad., oz. x.

M. S. A tablespoonful in water three times a day before meals.—*N. Y. Med. Record.*

